# Tramadol‐Induced Persistent Singultus: A Case Report on Diagnosis, Mechanism, and Management

**DOI:** 10.1002/ccr3.72964

**Published:** 2026-06-15

**Authors:** Ambika Nand Jha, Varsha Ratan Gaikwad, Abdullah Al Noman, Pranab Dev Sharma

**Affiliations:** ^1^ School of Pharmacy Sharda University Greater Noida Uttar Pradesh India; ^2^ Sandip School of Pharmaceutical Sciences Sandip University Nashik Maharashtra India; ^3^ School of Pharmacy BRAC University Dhaka Bangladesh; ^4^ Department of Biotechnology, School of Life Sciences BRAC University Dhaka Bangladesh

**Keywords:** baclofen therapy, chronic osteoarthritis, diaphragmatic spasm, opioid analgesia, persistent hiccups, singultus, tramadol‐induced adverse effect

## Abstract

Hiccups, or singultus, are involuntary diaphragmatic contractions followed by the sudden closure of the glottis, resulting in a characteristic sound. These episodes are generally transient but can be classified as acute (lasting < 48 h), persistent (lasting > 48 h), or intractable (lasting > 1 month). While most cases are self‐limiting, persistent or intractable hiccups often suggest an underlying organic, psychogenic, or idiopathic cause. Among the potential causes, drug‐induced singultus is a significant yet frequently underrecognized etiology. Although generally benign, persistent hiccups can cause substantial discomfort, disrupt sleep, and impair nutritional intake. In severe instances, they may lead to serious complications. Proper management relies on identifying the inciting factor and implementing appropriate therapeutic strategies. A 72‐year‐old male with a history of chronic right knee osteoarthritis was prescribed tramadol for pain management. Within 10 h of initiating tramadol therapy, the patient developed persistent singultus, characterized by repetitive involuntary diaphragmatic contractions at intervals of 10–15 s. This led to significant discomfort, impairing both his ability to rest and to consume food. Despite attempting non‐pharmacological interventions, such as drinking water and controlled respiration, the symptoms persisted. After a comprehensive assessment, tramadol was identified as the probable cause of the singultus. Consequently, the treatment plan was modified by discontinuing tramadol and introducing Baclofen, which resulted in complete symptom resolution. This case emphasizes the potential for tramadol to induce persistent singultus and underscores the importance of considering individual patient susceptibility when evaluating drug‐related adverse effects. Tramadol, while widely used for analgesia, can occasionally provoke rare adverse effects, including persistent singultus. The underlying pathophysiological mechanisms remain incompletely understood but may involve central nervous system pathways. Clinicians should exercise caution when prescribing tramadol, closely monitoring for potential adverse reactions to facilitate early identification and prompt intervention, thus minimizing patient discomfort and preventing further complications.

## Key Clinical Message

Tramadol, a widely utilized opioid analgesic, may precipitate persistent singultus as a rare yet clinically significant adverse effect. Early recognition, prompt drug discontinuation, and targeted pharmacological intervention are paramount for effective symptom resolution. Clinicians should maintain a vigilant approach to medication‐induced hiccups to optimize patient care and prevent unnecessary morbidity.

## 1 Introduction

Tramadol is a fully synthetic, centrally acting analgesic, chemically classified as a 4‐phenyl‐cyclohexanolamine derivative. It is indicated for the management of moderate to moderately severe pain [1, 2]. Its efficacy is derived from a multimodal mechanism involving the synergistic action of two enantiomers: (+)‐tramadol, which primarily inhibits serotonin reuptake, and (−)‐tramadol, which inhibits norepinephrine reuptake [3, 4, 5]. Both enantiomers, along with the active metabolite O‐desmethyltramadol (M1), are mainly responsible for its μ‐opioid receptor‐related analgesic effect [6, 7]. While Tramadol is clinically classified as a weak opioid possessing only about 10% of the analgesic potency of morphine, recent longitudinal studies demonstrate that it carries a comparable risk for long‐term dependency and substance use disorder [1, 2, 8, 9]. The clinical use of tramadol is frequently associated with gastrointestinal and central nervous system adverse effects, most notably nausea, dizziness, constipation, and somnolence (drowsiness) [1, 9]. While these symptoms are common to most opioids, tramadol's dual‐action mechanism results in a distinct safety profile; it carries a significantly higher incidence of nausea and vomiting compared to other analgesics, as well as unique risks for seizures, hypoglycemia, and serotonin syndrome [2, 3, 4, 7, 8].

Hiccups, which are known as singultus, occur when the diaphragm contracts repeatedly without control. Hiccups occur with the abrupt shutdown of the larynx [10]. For the most  part, hiccups resolve on their own. But when they last longer  beyond 48 h, they can disrupt sleep, hinder eating, produce stress, and lower quality of life [11]. Persistent or difficult‐to‐stop hiccups often signal another  problem in the body. These problems can be organic (related to the nervous  system) or due to medications [10, 11, 12]. Drug‐induced hiccups are known, but doctors frequently overlook them [13, 14]. Hiccups can result  from different types of medications, including corticosteroids, chemotherapy drugs, and  opioids [13, 15]. Tramadol is one of the most commonly consumed drugs in the  world. But there have been few reports in medical journals about  tramadol‐induced hiccups [16, 17]. The published reports are extremely sparse, consisting predominantly of case  reports and safety reviews. Therefore, the side effect is rare and poorly understood. For that reason, most physicians are unfamiliar with the tramadol‐induced hiccups. Moreover, there is no specific or normal procedure for the  diagnosis and treatment of tramadol‐induced hiccups.

Because very few cases have been reported and patients can suffer a lot, it is important to talk about tramadol‐related long‐lasting hiccups and how to manage them. We share this case to make doctors more aware of this rare side effect of the drug. We also suggest a simple way to manage it. The plan focuses on noticing the problem early and using the right medicines to bring fast relief from symptoms.

## 2 Case History and Examination

A 72‐year‐old retired civil engineer presented with persistent hiccups (singultus) following the initiation of tramadol for chronic right knee osteoarthritis. The patient had a longstanding history of osteoarthritis but no prior episodes of singultus or documented drug allergies. There was no history of hypertension, diabetes mellitus, cerebrovascular disease, epilepsy, or other neurological disorders. He had no known drug allergies and was not receiving any regular medications apart from tramadol. Within approximately 10 h of initiating tramadol therapy, the patient developed frequent, involuntary diaphragmatic contractions occurring every 10–15 s, which significantly interfered with his ability to eat and sleep.

On physical examination, the right knee showed no signs of inflammation, erythema, or effusion. The patient's vital signs were stable, and systemic evaluation revealed no abnormalities in the gastrointestinal, neurological, cardiovascular, renal, or metabolic systems. Neurological examination did not demonstrate any focal deficits, and the patient was fully conscious and oriented. Abdominal examination was unremarkable, with no distension or tenderness. Laboratory investigations, including a random blood glucose level (120 mg/dL) and serum creatinine (0.98 mg/dL), were within normal limits, effectively excluding metabolic or renal causes. The patient had no history of alcohol use or substance dependence. Given the clear temporal relationship between tramadol administration and the onset of symptoms, along with the absence of alternative clinical explanations, a preliminary diagnosis of tramadol‐induced singultus was considered.

## 3 Methods (Differential Diagnosis, Investigations, and Treatment)

A structured clinical approach was undertaken to identify the underlying cause of the persistent singultus. Given the close temporal relationship between symptom onset and tramadol use, a thorough differential diagnosis was formulated to exclude alternative etiologies. Possible causes included tramadol‐induced singultus, gastroesophageal reflux disease (GERD)‐associated hiccups, neurological disorders such as stroke, multiple sclerosis, or intracranial lesions, metabolic disturbances including electrolyte imbalances, uremia, or hyperglycemia, cardiovascular conditions like myocardial infarction or pericarditis, and other medication‐induced hiccups. However, tramadol was the only newly introduced medication, making it the most probable causative agent.

To exclude secondary causes, a detailed diagnostic assessment was performed. A comprehensive physical and systemic examination revealed no abnormalities in the gastrointestinal, neurological, or cardiovascular systems. Laboratory investigations, including blood glucose (120 mg/dL) and serum creatinine (0.98 mg/dL), were within normal limits, effectively ruling out metabolic disturbances. Neuroimaging was not performed as the patient had no focal neurological deficits, altered sensorium, headache, seizures, or signs suggestive of intracranial pathology. Clinical evaluation further reinforced the likelihood of tramadol‐induced singultus, as no other contributory factors were identified.

The patient's symptoms were managed using a structured therapeutic approach. Initial management involved the administration of intravenous ceftriaxone (1 g every 12 h) and intravenous omeprazole (20 mg every 12 h) to address potential infectious or acid‐related causes. Ceftriaxone was initiated empirically at presentation to cover a possible underlying infection while awaiting clinical clarification. However, the onset of singultus preceded ceftriaxone administration, and hiccups persisted despite continued ceftriaxone therapy. No temporal association was observed between ceftriaxone administration and symptom exacerbation or resolution, making it an unlikely confounding factor. Tramadol (100 mg intravenously every 12 h) was continued for pain relief. Despite ongoing treatment, the singultus persisted. Tramadol was then transitioned to an oral dose of 50 mg every 12 h, but hiccups recurred approximately one hour post‐administration. Non‐pharmacological measures, including breath‐holding techniques and controlled breathing exercises, were attempted but did not provide significant relief. As symptoms worsened, tramadol was discontinued on the fifth day. Baclofen (10 mg orally every eight hours) was initiated, leading to complete resolution of symptoms within three hours of tramadol cessation. Baclofen therapy was continued for three days, with no recurrence of singultus observed.

## 4 Conclusion and Results (Outcome and Follow‐Up)

This case highlights tramadol as a rare but clinically significant cause of persistent singultus. The rapid resolution of symptoms following tramadol discontinuation and baclofen initiation underscores the importance of early recognition and intervention in cases of drug‐induced hiccups. The patient experienced complete symptomatic relief within three hours of stopping tramadol, with no recurrence during the three‐day baclofen regimen. A one‐week follow‐up confirmed the sustained resolution of symptoms. The detailed clinical course, treatment approach, and patient response are outlined in Table 1.

**TABLE 1:** Clinical course, treatment, and response in a patient with tramadol‐induced singultus.

Day	Clinical presentation	Treatment administered	Response to treatment/outcome
Day 1	Onset of persistent singultus within 10 h of initiating intravenous tramadol therapy; repetitive diaphragmatic contractions at 10–15 s intervals, causing sleep and dietary disturbances.	IV tramadol 100 mg 12‐hourly, IV ceftriaxone 1 g 12‐hourly, IV omeprazole 20 mg 12‐hourly.	Hiccups persisted despite frequent water intake; no resolution of symptoms.
Day 2	Recurrent singultus episodes within 1 h of switching to oral tramadol 50 mg; associated with gastric bloating.	Oral tramadol 50 mg 12‐hourly, continued IV ceftriaxone and IV omeprazole.	Hiccups persisted for 2 h post‐administration; non‐pharmacological measures were ineffective.
Day 3	Persistent singultus episodes with increased frequency and duration; significant impact on sleep and daily activities.	Same regimen continued.	Temporary relief followed by recurrence after each tramadol dose.
Day 4	Singultus onset within 15 min of tramadol ingestion; duration increased to 3 h; considerable patient distress.	Same regimen continued.	No relief despite conservative measures; patient ceased non‐pharmacological interventions.
Day 5	Reporting of persistent singultus; tramadol discontinued; baclofen initiated.	Baclofen 10 mg oral 8‐hourly; tramadol discontinued.	Resolution of singultus within 3 h of tramadol withdrawal; baclofen was continued for 3 days without recurrence.

## 5 Discussion

Hiccups are involuntary contractions of the diaphragm, sometimes accompanied by the contraction of intercostal muscles and followed by the sudden closure of the glottis. Usually, they are harmless, but when they persist or become intractable, lasting for long periods, they can cause considerable discomfort and severely affect a patient's quality of life. Intractable hiccups, which last more than a month, are particularly concerning due to the symptoms they can cause, including sleep disturbances, fatigue in the respiratory muscles, malnutrition, and potentially dangerous complications like aspiration pneumonia or respiratory failure. Despite the fact that hiccups have been recognized for centuries, the exact mechanisms behind them are not fully understood.

The hiccup reflex is controlled by a complex neural pathway with three essential components: The afferent branch (involving the phrenic, vagus, and sympathetic nerves), the central unit (comprising the respiratory center, phrenic nerve nuclei, reticular formation, and hypothalamus), and the efferent branch, which activates the motor fibers of the diaphragm and intercostal muscles. Key neurotransmitters such as serotonin, dopamine, γ‐aminobutyric acid (GABA), glutamate, and glycine play important roles in regulating this reflex. Additionally, inputs from catecholaminergic and serotonergic afferents are involved in controlling the reflex's action [18, 19].

In this case, figuring out that tramadol was the cause meant carefully ruling out other common reasons for ongoing hiccups. A wide range of possible causes was looked at, including problems in the stomach and intestines, issues in the brain or nerves, changes in body chemistry, heart‐related problems, and side effects from medicines. Gastroesophageal reflux disease (GERD) was suspected first, and treatment with a proton‐pump inhibitor was tried, but it did not help at all. A full neurological check was done, and there were no signs of weakness, no abnormal findings, and nothing to suggest stroke or a brain mass. Blood tests were also normal, showing no electrolyte problems, no kidney issues like uremia, and no high blood sugar. Heart problems were thought about, too, but there was no chest pain, no shortness of breath, and the ECG looked normal, so that was unlikely. The patient did feel some bloating in the stomach for a short time, but the abdominal exam was completely normal. The most important detail was that tramadol was the only new medicine started before the hiccups began. The timing was very clear that the hiccups started within hours of taking tramadol and stopped after the drug was discontinued. Since no other clinical or lab findings pointed to another cause, this strong link makes tramadol‐induced hiccups the most reasonable diagnosis.

Tramadol, which is a medication that contains two enantiomers, has been linked to hiccups due to its impact on neurotransmitter systems. The cis‐enantiomer of tramadol binds to μ‐opioid receptors and blocks serotonin reuptake, potentially stimulating the central elements of the hiccup reflex. When tramadol binds to opioid receptors, it reduces GABA release, leading to an increase in dopamine. Furthermore, tramadol's active metabolite, O‐desmethyltramadol, has a greater affinity for μ‐opioid receptors, which further heightens the potential for hiccups. The trans‐enantiomer of tramadol inhibits norepinephrine reuptake and activates α2 receptors, which may further trigger the reflex and lead to diaphragmatic contractions [18, 20, 21].

Other factors, such as gastric distension, may also play a role in triggering hiccups. Gastric bloating can irritate the vagus nerve, activating the afferent signals of the hiccup reflex. This phenomenon was observed in our patient, who experienced bloating after taking tramadol, which could have contributed to the onset of hiccups [19, 22]. In this case, the close timing between tramadol administration and the onset of persistent hiccups strongly suggests a causal connection. Despite the use of non‐pharmacological interventions and symptomatic treatments, the patient's symptoms continued until tramadol was stopped and baclofen therapy was started. The resolution of symptoms after discontinuing tramadol (positive de‐challenge) and their return when tramadol was restarted (accidental rechallenge) further support the link between tramadol and the development of hiccups. This case highlights the importance of recognizing tramadol‐induced hiccups as a possible side effect, particularly in patients with gastrointestinal complaints, and underscores the need for timely intervention to prevent further complications.

To summarize, while tramadol‐induced hiccups are rare, they can have a significant impact on a patient's well‐being. The underlying mechanisms are multifaceted, involving the modulation of neurotransmitters, and can be aggravated by factors like gastric distension. Healthcare providers should be vigilant for this potential side effect, especially in patients who experience persistent or severe hiccups, and should consider alternative treatments such as baclofen to alleviate symptoms and improve patient quality of life.

## 6 Conclusion

The present case demonstrates that tramadol can be an uncommon but significant  etiology of persistent hiccups. If patients get new or  hard‐to‐control hiccups right after starting an opioid, doctors should consider tramadol as a possible cause, particularly when no other clear cause is evident. It's a big deal to detect it early, because stopping the drug  now can result in quick relief and save patients from additional tests or worry. In this case, baclofen was effective, and it may be useful as a significant alternative treatment when  hiccups do not resolve. From a medical perspective, verifying the medications as  well should be included in every workup for chronic hiccups. It may be possible to blame the tramadol, even if this side effect is  relatively rare. Recognizing this association can enable doctors to make the correct diagnosis and do more to keep  patients comfortable. More research is needed to  determine how frequently tramadol actually induces hiccups and what types of patients are most vulnerable. Future investigations, including  the prospectively planned trials and larger safety assessments that are underway, could offer insights as to why this side effect occurs and potentially inform improved therapeutic strategies.

## Author Contributions

**Ambika Nand Jha:** conceptualization, formal analysis, writing – original draft, writing – review and editing. **Varsha Ratan Gaikwad:** conceptualization, formal analysis, writing – original draft. **Abdullah Al Noman:** software, visualization, writing – original draft. **Pranab Dev Sharma:** software, visualization, writing – original draft, writing – review and editing.

## Funding

The authors have nothing to report.

## Ethics Statement

Prior to data collection, informed consent was obtained from the patient, ensuring they fully understood the study's purpose and procedures. The patient's confidentiality was guaranteed throughout the study, in strict adherence to ethical standards and privacy regulations.

## Consent

Written informed consent was obtained from the patient to publish this report. All authors have agreed to the submission and possible publication in the corresponding journal.

## Conflicts of Interest

The authors declare no conflicts of interest.

## Data Availability

Data sharing not applicable to this article as no datasets were generated or analysed during the current study.
